# Glypican-1 targeted antibody-based therapy induces preclinical antitumor activity against esophageal squamous cell carcinoma

**DOI:** 10.18632/oncotarget.15799

**Published:** 2017-03-01

**Authors:** Emi Harada, Satoshi Serada, Minoru Fujimoto, Yusuke Takahashi, Tsuyoshi Takahashi, Hisashi Hara, Rie Nakatsuka, Takahito Sugase, Takahiko Nishigaki, Yurina Saito, Kosuke Hiramatsu, Satoshi Nojima, Risa Mitsuo, Tomoharu Ohkawara, Eiichi Morii, Masaki Mori, Yuichiro Doki, Yasufumi Kaneda, Tetsuji Naka

**Affiliations:** ^1^ Laboratory of Immune Signal, National Institute of Biomedical Innovation, Health and Nutrition, Osaka, 567-0085, Japan; ^2^ Department of Gastroenterological Surgery, Osaka University Graduate School of Medicine, Osaka, 565-0871, Japan; ^3^ Department of Pathology, Osaka University Graduate School of Medicine, Osaka, 565-0871, Japan; ^4^ Division of Gene Therapy Science, Osaka University Graduate School of Medicine, Osaka, 565-0871, Japan

**Keywords:** esophageal squamous cell carcinoma, glypican-1

## Abstract

Esophageal squamous cell carcinoma (ESCC) has a poor prognosis despite the development of multimodal therapy. Expression of glypican-1 (GPC1) has been reported to be elevated in a subset of patients with ESCC and associated with chemoresistance. This study aimed to determine the association of GPC1 with ESCC growth and potential usefulness of the GPC1 targeted therapy by monoclonal antibody (mAb) in ESCC. Expression of GPC1 was higher in ESCC tumor tissues than in adjacent non-tumoral tissues and normal tissues. Knockdown of GPC1 decreased growth of ESCC cells and induced apoptosis via inhibition of EGFR, AKT and p44/42-MAPK signaling pathways *in vitro*. Anti-GPC1 mAb strongly inhibited tumor growth *via* antibody-dependent cellular cytotoxicity dependent and independent manner in GPC1-positive ESCC xenograft models. Anti-GPC1 mAb also inhibited tumor growth of GPC1 positive ESCC patients derived tumor xenograft models. Furthermore, anti-GPC1 mAb showed a significant tumor growth inhibition with decreased angiogenesis compared with IgG treated controls in ESCC xenografted mice. Treatment with anti-GPC1 mAb was not toxic in mice. Anti-GPC1 mAb may have a potent anti-tumor effect and represent a novel treatment option for patients with GPC1-positive ESCC.

## INTRODUCTION

Esophageal cancer is the sixth leading cause of cancer death worldwide and esophageal squamous cell carcinoma (ESCC) is the predominant histological type in Japan and Eastern countries [1]. Despite the development of multimodal therapy comprising surgery, chemotherapy and radiotherapy, the prognosis of ESCC remains poor [2]. To improve unfavourable outcomes of ESCC, it is important to explore the molecular mechanisms underlying ESCC and allow the development of urgently required novel therapeutic strategies.

Molecular targeted agents have become front-line cancer therapies. Molecular targeted therapy can act on various molecular pathways, including those involved in growth factor receptor signaling [epidermal growth factor receptor (EGFR) and Her-2/neu], the cell cycle, apoptosis and angiogenesis. In breast carcinoma, targeted therapy against HER2 using the humanized monoclonal antibody trastuzumab has now become integrated into standard adjuvant treatment regimens and has led to significant improvements in disease-free and overall survival in patients with Her2-positive cancer [3]. Therefore, the identification of potential cancer antigens for the development of innovative cancer-targeted therapies has become essential.

Recently, our group has identified glypican-1 (GPC1) as a novel cancer antigen for ESCC by quantitative proteomic approach focused on cell surface membrane protein [4]. Expression of GPC1 was elevated in most patients with ESCC and high expression levels of GPC1 were significantly associated with poor prognosis as well as chemoresistance [4]. GPC1 is a member of the glypican family of heparan sulphate proteoglycans (HSPGs) that are bound to the cell surface of the plasma membrane *via* glycosylphosphatidylinositol (GPI) linkages [5]. Several heparin-binding growth factors (HBGFs), including heparin-binding EGF-like growth factor (HB-EGF), fibroblast growth factor-2 (FGF-2) and hepatocyte growth factor (HGF) require HSPGs as co-receptors for efficient signalling [6]. GPC1 has been reported to enhance the interaction of several HBGFs with their specific receptors and modulate their biological activity [7]. Among these HBGFs, HB-EGF is a ligand of EGFR, is a member of the c-erb receptor family and is implicated in cell proliferation, differentiation and survival [8, 9]. Moreover, over-expression of EGFR has been observed in 50%–70% of ESCC tumors and is associated with poor prognosis [10, 11].

Our present study demonstrated that increased expression of GPC1 was associated with ESCC cell growth and survival by partially enhancing EGFR activity to suppress apoptosis. In addition, we developed an anti-GPC1 monoclonal antibody (mAb), which cross-reacts with mouse GPC1. Anti-GPC1 mAb induced significant tumor growth inhibition in ESCC xenograft models *via* antibody-dependent cellular cytotoxicity (ADCC) and complement-dependent cytotoxicity (CDC) dependent and independent manner. Importantly, anti-GPC1 mAb also induced potent tumor growth inhibition in GPC1 positive ESCC patient derived-tumor xenograft models. Furthermore, minimal toxicity was observed with anti-GPC1 mAb treatment in mice. These results suggest GPC1 may be a promising targeted therapy for ESCC.

## RESULTS

### Confirmatory expression analysis of GPC1 in human normal tissues and ESCC tumors

**Figure 1:**
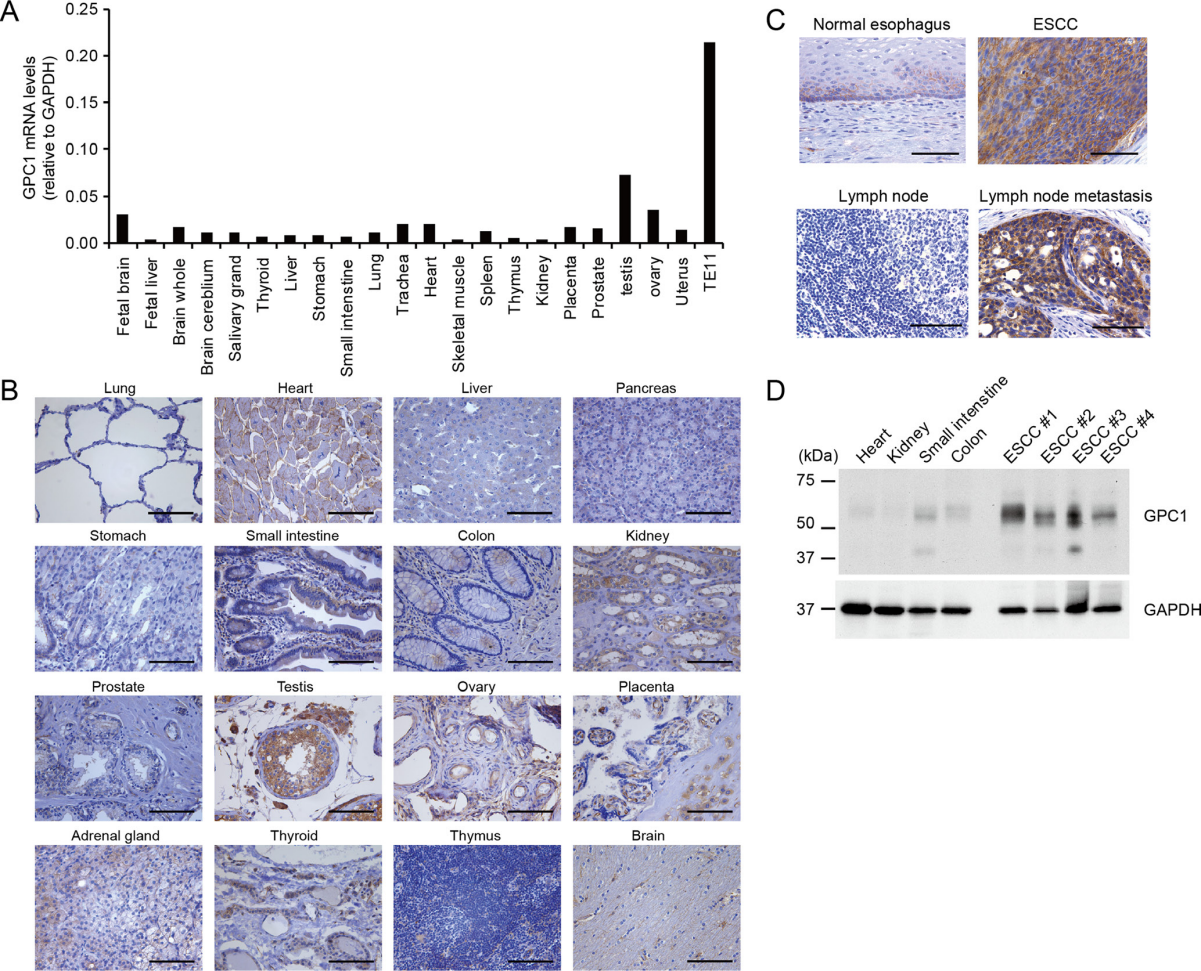
Confirmatory expression analysis of GPC1 in human normal tissues and ESCC tumors (**A**) Quantitative real-time PCR analyses were used to quantify GPC1 mRNA in various normal human tissues and GPC1 positive TE11 cells; GAPDH was used as an internal control. (**B**) Representative IHC GPC1 staining in normal tissues. Scale bar, 100 μm. (**C**) Representative IHC GPC1 staining in primary ESCC tissues, lymph node metastasis in ESCC and esophageal adenocarcinoma. Scale bar, 100 μm. (**D**) Western blot analysis of GPC1 in normal human heart, kidney, small intestine and ESCC tumors. Western blotting with anti-GPC1 antibody against proteins treated with heparinase III.

We have reported that expression of GPC1 was elevated in most patients with ESCC [4]. To evaluate the specificity of the expression of GPC1, we analysed expression profile of GPC1 in various normal tissues at mRNA levels by real time PCR analysis. We found that the expression levels of GPC1 were relatively low compared to TE11 cells, while slight expression of GPC1 was observed in testis, ovary and heart (Figure 1A).

Next, expression of GPC1 in normal tissues was evaluated by immunohistochemical (IHC) analyses using normal tissue microarray. Although GPC1 was strongly expressed in testis, GPC1 was weakly expressed in heart, kidney, ovary, placenta, adrenal gland and thyroid (Figure 1B). GPC1 expression was very weak or undetectable in lung, liver, pancreas stomach, small intestine, colon prostate, thymus and brain (Figure 1B). By western blotting, expression levels of GPC1 in human normal heart, kidney, small intestine and colon were weak compared to ESCC tissues (Figure 1D). As previously reported [4], IHC staining of GPC1 in tissue sections from patients revealed intense GPC1 staining in ESCC compared with that in normal esophageal tissue (Figure 1C). In addition, IHC analyses showed membranous immunoreactivity in ESCC cells, indicating GPC1 was localized to the cell surface. However, expression of GPC1 in normal esophagus was weak compared to ESCC (Figure 1C). In ESCC, lymph node metastasis is known to be strongly associated with poor prognosis [12]. Intriguingly, expression of GPC1 was also detected in lymph node ESCC metastases, indicating GPC1 may represent a therapeutic target for ESCC with lymph node metastasis (Figure 1C).These data indicate GPC1 may be an attractive therapeutic target for ESCC therapy.

### Knockdown of GPC1 expression induces growth inhibition of ESCC cells *in vitro*

**Figure 2:**
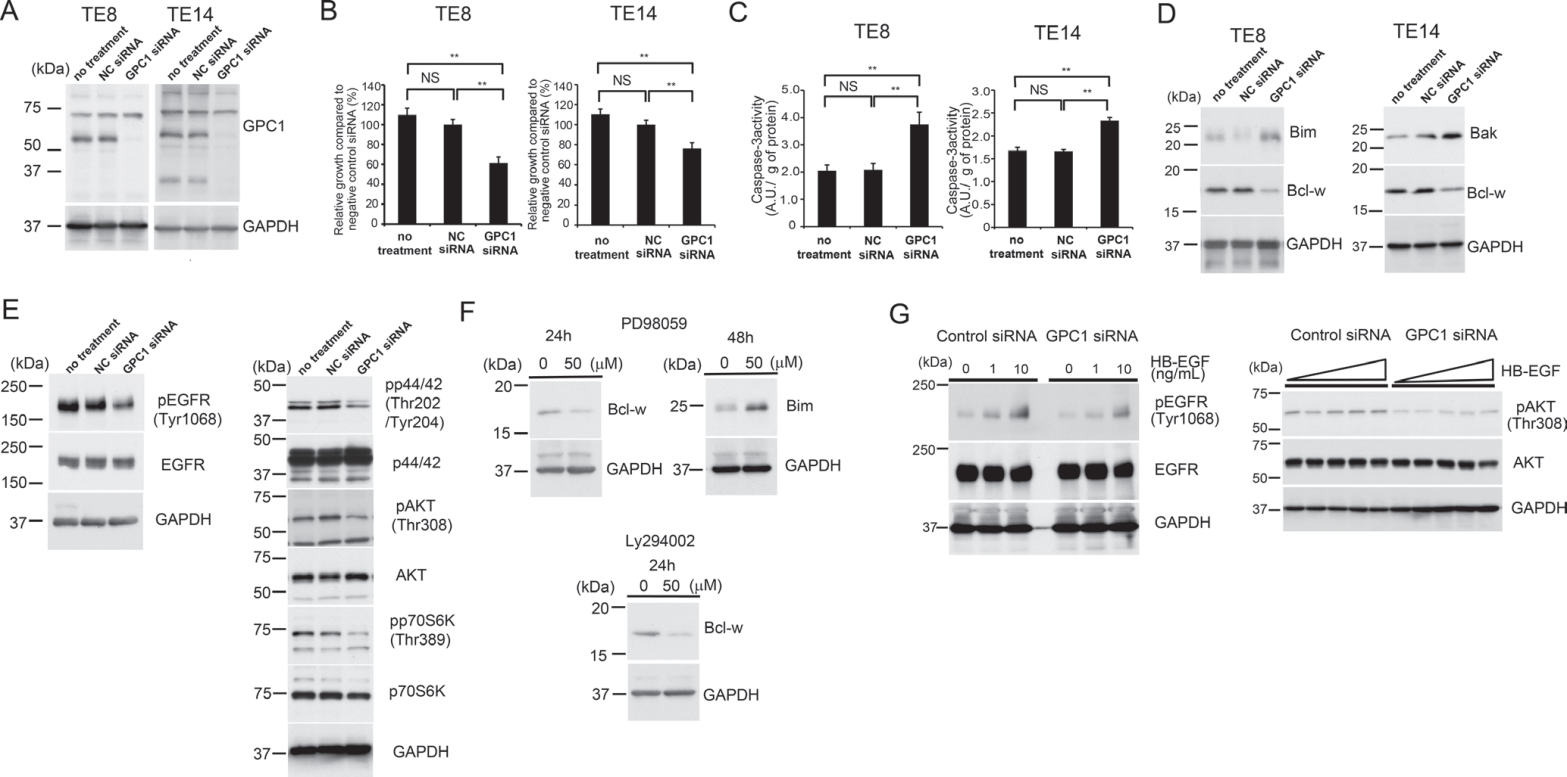
GPC1 is associated with ESCC proliferation by regulating AKT, p44/42 and EGFR signalling pathways (**A**) Knockdown of GPC1 expression by transfection with GPC1 siRNA. TE8 and TE14 cells were transfected with GPC1 or NC siRNA. Forty eight hours after transfection, knockdown of GPC1 was confirmed by Western blot analysis. (**B**) TE8 and TE14 cells were transfected with siRNA. Cell growth was assessed at 120 h using the WST-8 assay. Values were normalized to NC-siRNA-treated cells. (**C**) Caspase-3 activities were measured 72 h after siRNA transfection. (**D**) Knockdown of GPC1 increased expression of pro-apoptotic proteins and decreased expression of anti-apoptotic proteins. TE8 and TE14 cells were transfected with GPC1 siRNA or NC siRNA for 48 h. Extracted proteins were immunoblotted with indicated antibodies. (**E**) Constitutive activation of EGFR, AKT and p44/42-MAPK signalling pathways was inhibited by knockdown of GPC1. TE8 cells were transfected with GPC1 siRNA or NC siRNA. After 48 h, protein extracts were immunoblotted with indicated antibodies. (**F**) Increased expression of pro-apoptotic proteins and decreased expression of anti-apoptotic proteins was observed after treatment with PD98059 or Ly294002 for 24 or 48 h in TE8 cells. Protein extracts were immunoblotted with indicated antibodies. (**G**) GPC1 enhances activation of EGFR by HB-EGF. TE8 cells were transfected with GPC1 siRNA or NC siRNA for 48 h. (Left panel) Cells were stimulated with 0, 1.0, 10 ng/mL HB-EGF for 10 min. (Right panel) After serum starvation for 3 h, cells were stimulated with 0, 1.25, 2.5, 5, 10 ng/mL HB-EGF for 15 min. Protein extracts were immunoblotted with indicated antibodies.

To examine whether GPC1 expression contributes to growth of ESCC cells, the effect of GPC1-siRNA treatment in two ESCC cell lines expressing GPC1 (TE8 and TE14) as previously reported [4], was evaluated using the WST-8 assay. To ensure silencing efficiency, decreased expression of GPC1 was analysed by Western blot analysis 48 h after siRNA transfection (Figure 2A). There was marked inhibition of cell growth in GPC1-siRNA-transfected cells compared with negative control (NC)-siRNA-treated cells and untreated cells in each ESCC cell line (Figure 2B). In TE8 and TE14 cells treated with GPC1 siRNA, markedly increased caspase-3 activity was detected (Figure 2C). In addition, knockdown of GPC1 in TE8 and TE14 cells decreased levels of the anti-apoptotic protein Bcl-w, and increased levels of the pro-apoptotic proteins Bim in TE8 cells and Bak in TE14 cells (Figure 2D), suggesting that suppression of GPC1 protein expression by siRNA resulted in increased apoptosis in these ESCC cell lines.

### Knockdown of GPC1 expression inhibits EGFR, AKT and p44/42-MAPK signalling pathways in ESCC cells *in vitro*

Previously, it has been reported that there is an association between EGFR signalling and ESCC progression, and several types of EGFR targeted therapy were recently developed [13, 14]. GPC1 has been shown to be a co-receptor of various HBGFs, including HB-EGF, amphiregulin, HRG and FGF-2, which promote biological activity [15]. To elucidate proliferation-associated signalling pathways regulated by GPC1 in ESCC cells, phosphorylation levels of EGFR were analysed by Western blot analysis. Notably, phosphorylation levels of EGFR (Tyr1068) were decreased in cells transfected with GPC1 siRNA compared with NC-siRNA-treated cells and untreated cells (Figure 2E). AKT and p44/42-MAPK signalling pathways are major downstream effectors of the EGFR signalling pathway associated with survival and proliferation of cancer cells. In accordance with the inhibition of phospho-EGFR (Tyr1068), knockdown of GPC1 also decreased levels of phospho-AKT (Thr308), phospho-p70S6K (Thr389) and phospho-p44/42-MAPK (Thr202/Try204) in TE-8 cells (Figure 2E). In addition, treatment with the MEK1 inhibitor PD98059 resulted in increased expression of Bim and decreased expression of Bcl-w (Figure 2F) or the PI3 kinase inhibitor Ly294002 resulted in decreased expression of Bcl-w (Figure 2F), consistent with results obtained from GPC1 siRNA transfection studies.

### Knockdown of GPC1 expression inhibits activation of EGFR *via* HB-EGF in ESCC cells *in vitro*

We assessed the effects of the GPC1 knockdown on the activation of EGFR by stimulation with HB-EGF. Compared with the control siRNA-transfected TE8 cells, knockdown of GPC1 resulted in decreased activation of phospho-EGFR (Tyr1068) following stimulation with HB-EGF (Figure 2G left panel). In addition, AKT phosphorylation in response to stimulation with HB-EGF was attenuated by transfection with GPC1 siRNA compared with NC siRNA in transfected TE8 cells (Figure 2G right panel), suggesting that GPC1 regulates EGFR activity and downstream AKT signalling induced by HB-EGF.

### Production of anti-GPC1 mAb

**Figure 3:**
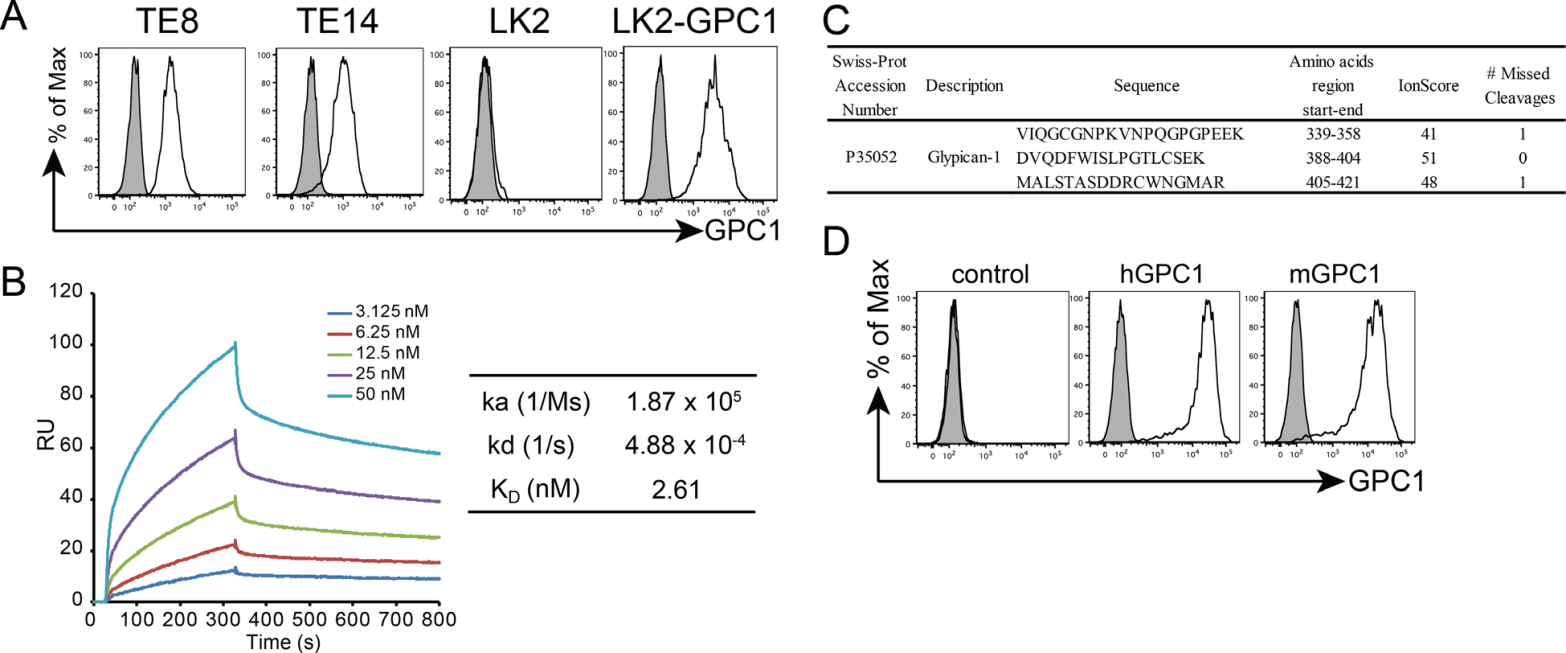
Isolation and characteristics of human antibody targeting GPC1 (**A**) Flow cytometry of antibody binding to GPC1-positive (TE8, TE14), GPC1-negative (LK2) and cells stably transfected with the LK2 gene encoding GPC1 (LK2-GPC1). The shaded area histogram profile indicates the isotype control. Open histogram indicates anti-GPC1 mAb staining. (**B**) SPR analysis of anti-GPC1 mAb. (**C**) Epitope analysis of anti-GPC1 mAb by mass spectrometry. Recombinant human GPC1 proteins were mixed with anti-GPC1 mAb or mouse IgG2a and digested with trypsin. After digestion, immune complexes were immunoprecipitated with protein G-Sepharose. Eluted peptides were reduced, alkylated and analyzed by LC–MS/MS. (**D**) Anti-GPC1 mAb clone 1-12 cross-reacted with mGPC1. HEK293 cells were transfected with empty vector, full length hGPC1 or mGPC1 expression vectors. The shaded histogram profile indicates control samples without primary antibody staining and the open histogram indicates results of anti-GPC1 antibody staining.

Because GPC1 is frequently over-expressed in ESCC and associated with increased growth in ESCC, we attempted to develop an antibody-based therapy targeting GPC1. Because hGPC1 and mouse GPC1 (mGPC1) proteins are highly homologous (88.71% sequence identity), hGPC1 likely has little antigenicity in mice. We therefore used chickens as hosts for antigen immunization. Single-chain variable fragment (scFv) clones positively bound to GPC1 were selected and chicken/mouse chimeric mAb with mouse IgG2a Fc domains were generated as mouse IgG2a mediates high levels of ADCC and CDC activity [16]. We then tested the affinity of the chicken/mouse chimeric anti-GPC1 mAb in two native ESCC cell lines (TE8 and TE14), one GPC1-negative lung squamous carcinoma cell line (LK2) and an LK2-derived cell line (LK2-hGPC1) by flow cytometry. The LK2-hGPC1 cell line expresses stable and high levels of cell surface GPC1. Chicken/mouse chimeric anti-GPC1 mAb (clone 1–12) demonstrated specific binding to ESCC cells and LK2-hGPC1 cells but not to GPC1-negative LK2 cells (Figure 3A). We measured the binding affinities of chicken/mouse chimeric anti-GPC1 mAb to GPC1 protein by SPR analysis. The calculated K_D_ value was 2.61 nM for clone 1–12, comparable with the affinities of approved cancer therapeutic antibodies (Figure 3B).

### Epitope mapping, cross-reactivity and indirect cytotoxity assay of anti-GPC1 mAb

Mass spectrometry was used to determine the epitope region of the anti-GPC1 mAb. hGPC1 protein was incubated with anti-GPC1 mAb or control mouse IgG2a and digested with trypsin. After immunoprecipitation with protein G-Sepharose, antibody-bound peptides were eluted and amino acid regions were identified by LC–MS/MS analysis. GPC1-derived peptides 339–358, 388–404 and 405–421 were specifically identified as anti-GPC1 mAb-bound peptide compared with control mouse IgG2a (Figure 3C).

To determine whether anti-GPC1 mAb cross-reacted with mGPC1, HEK293 cells were transfected with an mGPC1 expression vector. In addition to showing affinity for hGPC1, we found anti-GPC1 mAb also reacted with mGPC1 by flow cytometry (Figure 3D).

### Toxicology of anti-GPC1 mAb

Because anti-GPC1 mAb cross-reacted with mGPC1, *in vivo* toxicology studies were performed in C57BL/6 mice to examine the toxicity of anti-GPC1 mAb. Treatment with anti-GPC1 mAb antibody at a dose of 50 mg/kg did not cause significant changes in serum chemistry or blood cell counts after 7 days compared with mice treated with IgG2a control antibodies (Supplementary Tables 1, 2). No histologic changes in liver, lung, heart, kidney, spleen, brain and testis were observed following treatment with anti-GPC1 mAb (Supplementary Figure 1).

### Anti-GPC1 mAb induces anti-tumor activity *in vivo*

**Figure 4:**
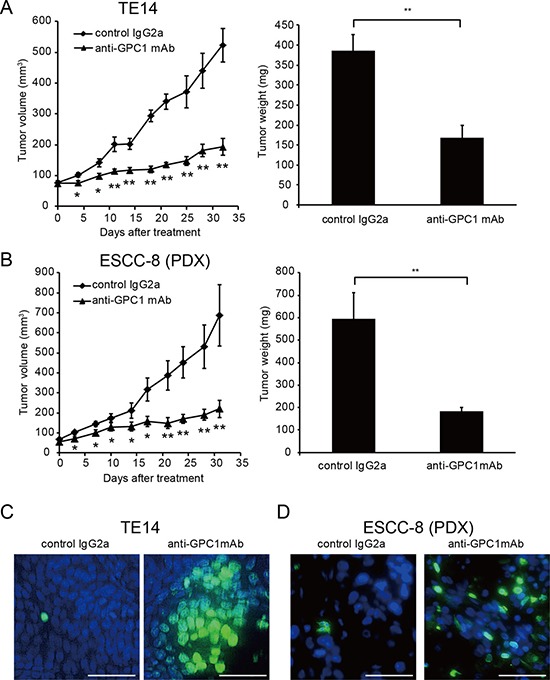
Anti-GPC1 mAb inhibits ESCC growth in SCID mice xenografted with GPC1 positive ESCC cell line and ESCC tissues *in vivo* (**A**) Anti-tumor activity of anti-GPC1 mAb against TE14 xenografts. SCID mice were subcutaneously inoculated with 2 × 10^6^ cells. Mice were xenografted with GPC1-positive (TE14) and divided into two groups (5–6 mice/group) when tumor volumes reached an average size of 70 mm^3^. Mice were intraperitoneally treated with 10 mg/kg anti-GPC1 mAb or control mouse IgG2a. Thirty two days (TE14 cells) after first treatment, tumors were removed and weighed. (**B**) Anti-tumor activity of anti-GPC1 mAb against ESCC-8 patients tumor xenografted mice. SCID mice were subcutaneously inoculated with ESCC-8 tumor tissues. Mice were divided into two groups (7 mice/group) when tumor volumes reached an average size of 70 mm^3^, and mice were intraperitoneally treated with 10 mg/kg anti-GPC1 mAb or control mouse IgG2a.Thirty one days after first treatment, tumors were removed and weighed. *P < 0.05, **P < 0.01. (**C**, **D**) TUNEL assay (blue fluorescence = DAPI staining for nuclei; cyan fluorescence = TUNEL positivity) in TE14 (C) or ESCC-8 PDX tissues from animals treated with control mouse IgG2a or anti-GPC1 mAb. Scale bar = 50 mm.

To evaluate the antitumor activity of anti-GPC1 mAb in animals, SCID mice were subcutaneously inoculated with TE14 cells and then intraperitoneally treated with 10 mg/kg anti-GPC1 mAb twice-weekly for three weeks. Compared with isotype control mouse IgG2a, administration of anti-GPC1 mAb significantly inhibited the growth of the TE14 xenografts assessed by tumor volume (60.99% ± 5.11% tumor growth inhibition at day 32) and tumor weight (Figure 4A).

Next, we also assessed the anti-tumor effect of anti-GPC1 mAb against ESCC patient tumor-derived xenograft (PDX) model (designated ESCC-8). ESCC tissues were subcutaneously implanted to the NOG mice. Furthermore, tumor tissues were subcutaneously implanted to the SCID mice to assess the anti-tumor effect of anti-GPC1 mAb. In this ESCC-8 PDX model, expression of GPC1 in the tumor tissue was confirmed by IHC analysis (Supplementary Figure 2). Anti-GPC1 mAb significantly inhibited the growth of the ESCC-8 PDX compared with isotype control mouse IgG2a (67.87% ± 6.28% tumor growth inhibition at day 31) and also tumor weight (Figure 4B). By TUNEL staining, we detected marked apotosis in tumors of anti-GPC1 mAb treated mice compared to control IgG treated mice in both TE14 xenograft and ESCC-8 PDX models (Figure 4C and 4D).

**Figure 5:**
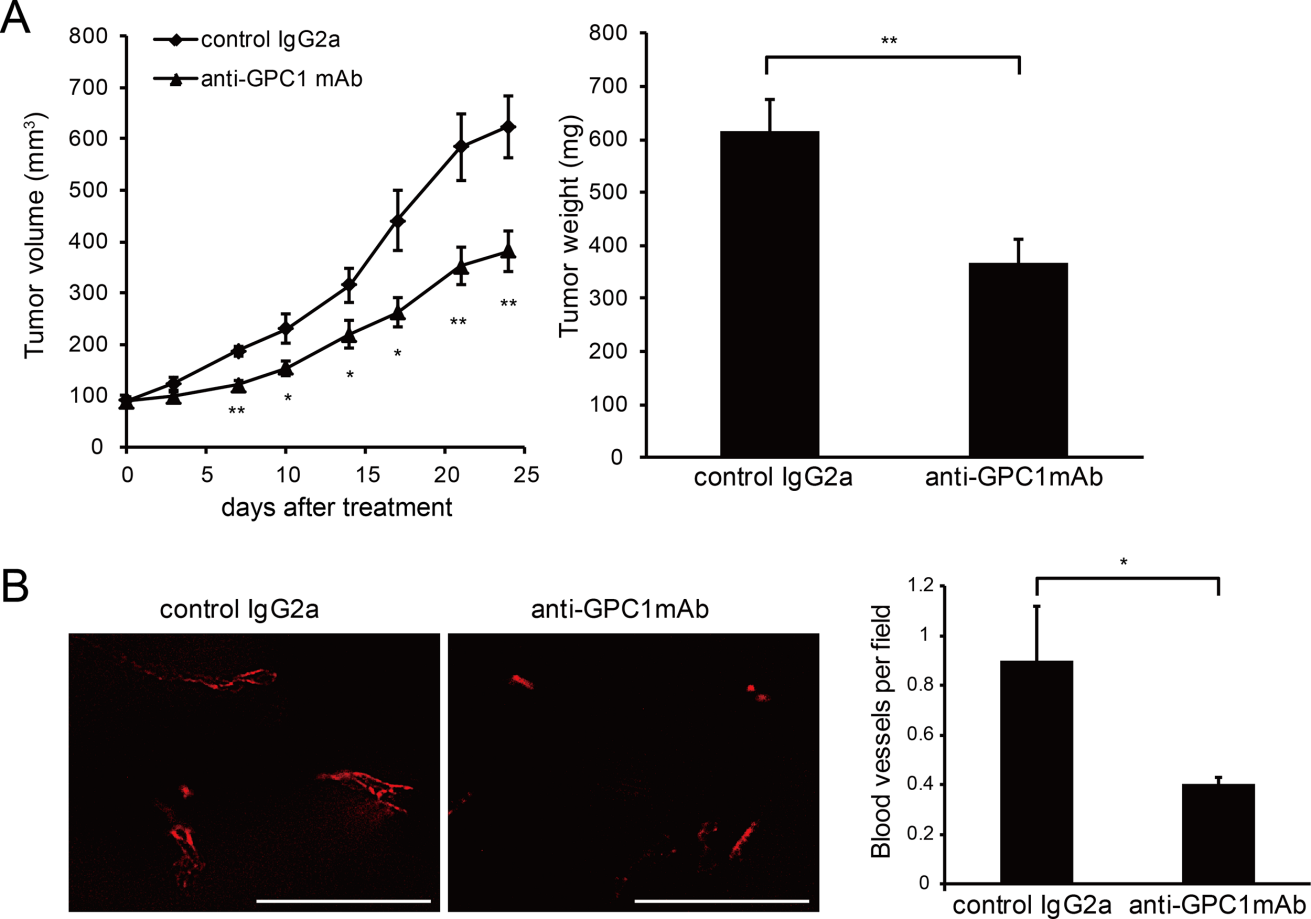
Anti-GPC1 mAb inhibits ESCC growth in NOD/SCID mice *in vivo* Anti-tumor activity of anti-GPC1 mAb against TE14 in NOD/SCID mice. (**A**) NOD/SCID mice were subcutaneously inoculated with 2 × 10^6^ TE14 cells. When tumor volumes reached an average size of 70 mm^3^, mice were intraperitoneally treated with 10 mg/kg anti-GPC1 mAb or control mouse IgG2a. Twenty four days after first treatment, tumors were removed and weighed. (**B**) Immunohistochemical staining of CD31 (red) in TE14 xenografts NOD/SCID mice and quantification of CD31 positive area. Scale bar, 100 μm. *P < 0.05, **P < 0.01.

NOD/SCID mice, known to have low natural killer cell activity and no CDC activity and functional B and T cells, were used to assess the contribution of ADCC and CDC to the anti-tumor effect of anti-GPC1 mAb. Notably, while anti-GPC1 mAb seemed less effective in NOD/SCID mice than in SCID mice, it still showed tumor growth inhibition (38.7% ± 6.39) in TE14 xenografts in NOD/SCID mice compared with control mice treated with mouse IgG2a (Figure 5A). We found that GPC1 was expressed in vascular endothelium cells in ESCC tumor (Supplementary Figure 3), and tumor angiogenesis might be inhibited by anti-GPC1 mAb *in vivo*. By CD31 staining, we observed significantly decreased blood vessels in anti-GPC1 mAb treated mice compared to control IgG2a (Figure 5B). These results suggest that anti-GPC1 mAb inhibits tumor growth *in vivo* in ADCC and CDC dependent and independent manners including inhibition of tumor angiogenesis.

## DISCUSSION

In this study, we demonstrated GPC1 as a suitable ESCC cancer antigen for antibody-based therapy. GPC1 was associated with increased proliferation of ESCC through inhibition of apoptosis (Figure 2). GPC1 was also found to be involved in the activation of the EGFR signalling pathway in response to EGFR ligands including HB-EGF, which is known to be one of the HBGFs. Importantly, we have recently demonstrated that high tumoral expression of GPC1 in ESCC, determined by IHC analysis, was significantly associated with poor prognosis compared to low expression of GPC1 and higher expression of GPC1 associated with elevated chemoresistance to cisplatin. We also demonstrated that anti-GPC1 mAb (clone 1–12) induced marked tumor growth inhibition in GPC1-positive human ESCC xenograft models. Considering that the expression of GPC1 in normal tissue is limited, targeting GPC1 by anti-GPC1 mAb would be a promising therapy for ESCC expressing GPC1.

It has previously been reported that GPC1 is involved in cell cycle regulation [17–19]. Qiao *et al*. demonstrated ectopic expression of GPC1 stimulates S phase entry *via* downregulation of tumor suppressors, including pRb and Cip/Kip cyclin-dependent kinase inhibitors, and upregulation of pro-oncogenic proteins, including cyclin E and cyclin-dependent kinase 2, in the human glioblastoma cell line U87-MG [19]. Silencing of GPC1 expression by siRNA transfection induced G0/G1 growth arrest in TE8 and TE14 cells (Supplementary Figure 4). However, the role of GPC1 in the regulation of apoptosis has not previously been reported. As shown in Figure 2C, siRNA-mediated knockdown of GPC1 resulted in a significant level of apoptosis in TE8 and TE14 cells *via* induction of increased expression of the pro-apoptotic proteins Bim and Bik and decreased expression of the anti-apoptotic protein Bcl-w. These data indicate a critical role of GPC1 in the growth and survival of ESCC cells through regulation of apoptosis in addition to the cell cycle.

We produced chicken/mouse chimeric mAb against human GPC1, which cross-reacts with mGPC1, and demonstrated marked tumor growth inhibition by anti-GPC1 mAb in SCID mice xenografted with GPC1-positive TE14 cells *via* ADCC and CDC activity. Intriguingly, anti-GPC1 mAb also represented potent antitumor effect against GPC1 positive ESCC PDX model, suggesting that the potential usefulness of this mAb in patients with GPC1 positive ESCC. In addition, anti-GPC1 mAb also partially inhibited tumor growth in NOD/SCID mice, inhibiting tumor angiogenesis (Figure 5B), suggesting that the possibility of the presence of neutralizing activity of GPC1 in this clone. Notably, GPC1 has previously been reported to be expressed on tumor vascular endothelial cells but not on normal vascular endothelial cells [20]. Furthermore, it has been reported that host-derived GPC1 in addition to tumoral GPC1 is involved in tumor neovascularization [21]. Considering that vascular endothelial growth factor and basic fibroblast growth factor, both of which are known as HBGFs, are important factors for the proliferation of vascular endothelial cells, anti-GPC1 mAb may inhibit tumor growth *in vivo* by blocking GPC1 as a co-receptor activity of these factors against vascular endothelial cells and inhibiting neovascularization, although further studies are required to completely elucidate this potential mechanism.

In our previous study [4], by IHC immunohistochemical assessment of 175 ESCC specimens, 98.8% expressed GPC1. We further showed that high GPC1 expression is associated with poor prognosis by Kaplan-Meier survival analysis and increased chemo-resistance by clinicopathological analysis. In addition, we also showed that GPC1 expression *in vitro* enhances chemo-resistance of ESCC to cisplatin not by increasing transport and excretion of drugs but by antagonizing apoptosis through upregulating MAPK signaling and Bcl-2 family signaling. These results indicate that GPC1 positive tumor cells are less sensitive to cisplatin. Since anti-GPC1 mAb specifically target GPC1 positive tumor cells, we consider that anti-GPC1 mAb would be quite effective therapy for treatment of ESCC cells which remain after cisplatin therapy.

The amino acid sequences of epitope regions recognized by anti-GPC1 mAb (clone 1–12) were highly conserved in hGPC1 and mGPC1. Because anti-GPC1 mAb cross-reacts with mGPC1, we assessed toxicity in mice treated with anti-GPC1 mAb and found no evidence of blood biochemistry changes or histological abnormalities, including esophagus and testis, demonstrating the low toxicity and therapeutic potential of anti-GPC1 mAb. We observed over-expression of GPC1 in primary ESCC tissue (Figure 1B), whereas expression of GPC1 was limited in other tissues such as the testis. Moreover, GPC1 was found to be expressed in lymph node metastases (Figure 1B), suggesting that systemic treatment with anti-GPC1 mAb may also have efficacy in patients with lymph node ESCC metastases. Further analyses are warranted regarding distant metastasis because the expression of GPC1 in distant metastasis was not examined in this study due to the difficulty in obtaining clinical samples. In addition to ESCC, increased expression of GPC1 has been reported in pancreatic cancer, breast cancer and glioma and shown to promote the mitogenic, metastatic and angiogenic properties of cancers [6, 21–24]. Therefore, anti-GPC1 mAb may have anti-tumor efficacy in these GPC1-positive cancers. We are currently conducting studies evaluating the anti-tumor efficacy of anti-GPC1 mAb in xenograft models using cancer types other than ESCC.

In conclusion, anti-GPC1 mAb inhibits ESCC growth by targeted inhibition of GPC1. Our data suggest that GPC1 is associated with tumor growth and that targeting GPC1 with anti-GPC1 mAb represents a therapeutic strategy to decrease tumor growth in patients with GPC1-positive ESCC.

## MATERIALS AND METHODS

### Tissue samples

Written informed consent was obtained for all cases and the experimental protocol was approved by the ethics committees of Osaka University and National Institute of Biomedical Innovation, Health and Nutrition.

### Cell lines

Human esophageal squamous cancer cell lines (TE8, TE11 and TE14) were obtained from the RIKEN BioResource Center. A human lung squamous cancer cell line (LK2) was obtained from the Japanese Collection of Research Bioresources (Osaka, Japan). These cell lines were cultured in RPMI 1640 medium (Wako Pure Chemical Industries) supplemented with 10% FBS (Serum Source International, NC, USA), 100 U/ml penicillin and 100 μg/ml streptomycin (Nacalai Tesque, Kyoto, Japan). Establishment of GPC1 stable transfectant LK2 cell lines were described previously [4], and cell lines were cultured in RPMI 1640 medium supplemented with 10% FBS and 250 μg/ml G418 (Life technologies, Carlsbad, CA,USA). These cell lines were cultured at 37°C under a humidified atmosphere of 5% CO_2_. The identity of each cell line was confirmed by DNA fingerprinting *via* short tandem repeat profiling, as previously described [25].

### Reagents and antibodies

PD98059 and Ly294002 were purchased from Cell Signaling Technology (Danvers, MA). Recombinant human HB-EGF was purchased from R&D Systems (Minneapolis, MN). The following primary antibodies were used: anti-GPC1 antibody from Atlas antibodies (Stockholm, Sweden); anti-phospho-Akt (Thr308), anti-Akt, anti-phospho-p70S6K (Thr389), anti-p70S6K, anti-phospho-p44/42 (Thr202/Tyr204), anti-p44/42, anti-Bak, anti-Bim, anti-Bcl-w and anti-phospho-EGFR (Tyr1068) from Cell Signaling Technology; anti-GAPDH from Santa Cruz Biotechnology (Santa Cruz, CA) and anti-EGFR from BD Transduction Laboratories (San Jose, CA).

### Quantitative reverse transcription-PCR (qRT-PCR) analysis

Human MTC Panel I and Human MTC Panel II (Clontech, Palo Alto, CA, USA) were used as a source cDNA from several normal human tissues. For positive control total RNA was extracted from TE11 cells and cDNA was prepared as described previously [25]. To confirm expression of GPC1, qRT-PCR was performed as previously described [25]. GAPDH was used as a housekeeping gene for quantitative real-time PCR normalization. Primer sequences used were as follows: GPC1, forward primer 5′-GCCAGATCTACGG AGCCAAG-3′ and reverse primer 5′-AGGTTCTCCTCC ATCTCGCT-3′ and GAPDH, forward primer 5′- AGCA ATGCCTCCTGCACCACCAAC-3′ and reverse primer 5′- CCGGAGGGGCCATCCACAGTCT-3′.

### Digestion with heparinase III

Proteins were extracted from fresh-frozen samples of ESCC with 50 mM Tris-HCl (pH 7.4),150 mM NaCl, 1 mM EDTA, 1% Triton X-100, 1% protease inhibitor cocktail (Nacalai Tesque) and 1% phosphatase-inhibitor cocktail (Nacalai Tesque). The total proteins from adult human normal heart, kidney, small intestine, colon tissues were purchased from BioChain Institute, Inc (Hayward,CA, USA). Protein extracts from ESCC tissues or normal tissues were digested with Heparinase III, as described previously [6], and used for western blotting.

### Western blotting

Cells were lysed in radioimmunoprecipitation assay buffer (10 mM Tris-HCl, pH 7.5, 150 mM NaCl, 1% Nonidet P-40, 0.5% sodium deoxycholate, 0.1% SDS, 1% protease-inhibitor cocktail and 1% phosphatase-inhibitor cocktail). Following centrifugation (13,200 rpm, 4°C, 15 min), soluble proteins in the supernatant were separated using SDS-PAGE, as previously described [26].

### Immunohistochemistry

Surgically resected tumor tissues were obtained from patients with ESCC from Osaka University Hospital (Osaka, Japan). Detailed methods are described in the online Supplementary Material.

Frozen sections were prepared from tumor tissues and stained for CD31 using a rat anti-mouse CD31 (BD Biosciences) followed by the Alexa-647-conjugated second antibody. Fluorescence images were captured using Biozero BZ-9000 (Keyence, Tokyo, Japan) in five random fields at 400× magnification. The fluorescence was quantitated by a standardized procedure using a BZ-II Analyser (Keyence).

### Small interfering RNA transfection

Commercial human GPC1 (hGPC1) small interfering RNA (siRNA) and negative control siRNA were obtained from QIAGEN (Valencia, CA, USA). Cells were transfected with siRNA using Lipofectamine 2000 reagent (Invitrogen), according to the manufacturer's instructions. For gene silencing, a specific sense strand (5′-gggacacgcucacggccaatt-3′) was used for hGPC1 siRNA, and an antisense strand (5′-uuggccgugagcguguccctg-3′) was used as a control. Selective silencing of hGPC1 was confirmed by Western blot analysis.

### Cell growth assay

ESCC cells were plated in 96-well plates (1,000 cells/well for TE8 or 2,000 cells/well for TE14) and grown in their respective media for 120 h after siRNA transfection. At each time point, cell growth was assessed using the WST-8 assay as previously described [25].

### Apoptosis assay

ESCC cells were seeded in 6-well plates at a density of 2 × 10^5^ cells per well, and transfected with siRNA targeting hGPC1 or NC siRNA for 72 h. The cells were washed with PBS, and caspase-3 activity was detected using the caspase-3 fluorometric assay kit (R&D systems) according to the manufacturer's instructions. The presented values are representing the means of three independent experiments.

### Antibody production, epitope and cross-reactivity analysis of anti-GPC1 mAb

Detailed methods are described in the online Supplementary Material.

### Fluorescence activated cell sorting (FACS) analysis

Cells were washed twice in PBS (Nacalai Tesque) and detached with 0.02% EDTA solution (Nacalai Tesque). Cells were washed twice with cold FACS buffer (PBS supplemented with 1% FBS and 0.1% sodium azide) and then incubated with chicken/mouse chimeric anti-GPC1 antibody (clone 1–12) at a 1:100 dilution and labelled with FITC-labelled goat anti-mouse IgG (H + L chain specific) antibody (SouthernBiotech, Birmingham, AL, USA). Stained cells were analysed using a FACS Canto II cytometer (Becton Dickinson, Mountain View, CA, USA) and the results were analysed using FlowJo software (Tree Star, Stanford, CA, USA).

### Surface plasmon resonance (SPR) analysis

The binding affinity of each anti-hGPC1 antibody to hGPC1 was assessed by SPR using BIAcore3000 (GE Healthcare UK Ltd., Chalfont, United Kingdom). Detailed methods are described in the online Supplementary Material.

### *In vivo* toxicology studies

C57B/6 mice at 8 weeks were administered with anti-GPC1 mAb (i.p. 50 mg/kg). Isotype control IgG (mouse IgG2aκ, Sigma) was used as a control. One week after administration, (3 mice/group) complete blood counts and serum chemistry were analysed and dissected organs were pathologically evaluated by H&E staining.

### Tumor xenograft and antibody therapy

Healthy female CB17/severe combined immunodeficient (SCID) mice and non-obese diabetic (NOD)/SCID mice at 6-week-old were obtained from Charles River Japan (Yokohama, Japan). Animals were maintained in a specific pathogen-free facility. For xenograft experiments, SCID and NOD/SCID mice were inoculated subcutaneously with 2 × 10^6^ TE14 cells in a total volume of 100 μl of 1/1 (v/v) PBS/Matrigel (BD Biosciences) into the flank. When the mean tumor sizes reached approximately 70 mm^3^ for TE14, mice were then randomly divided into two groups (six mice/group) and isotype control IgG (mouse IgG2aκ, Sigma) or chicken/mouse chimeric anti-human GPC1 mAb (clone 1–12, Fc type is mouse IgG2a) was administered at a dose of 10 mg/kg in 400 μl of PBS twice-weekly for 3 weeks. Tumor sizes were measured twice-weekly using vernier callipers throughout the study. Tumor volumes were determined by measuring two dimensions, length (*L*) and width (*W*) and calculating the volume as (*W*^2^ × *L*)/2. Tumors were resected and weighed 32 days (TE14 cells) after first treatment. All animal experiments were conducted according to the institutional ethical guidelines for animal experimentation of the National Institute of Biomedical Innovation, Health and Nutrition.

### Development of patient tumor-derived xenograft (PDX) mouse models and antibody therapy

Healthy female NOD/Shi-scid-IL2Rγ null (NOG) mice at 6-week-old were purchased from Central Institute for Experimental Animals (Kawasaki, Japan). Animals were maintained at the local animal facility according to the legislation and ethical approval was obtained for the establishment of PDX. Use of human tissues was permitted by the ethics committees of the Osaka University, Graduate School of Medicine and the National Institute of Biomedical Innovation, Health and Nutrition. The ESCC8-PDX mouse models were established with fresh ESCC tissues endoscopically resected from ESCC patients. Briefly, endoscopically resected patient's ESCC tissues (P0 tissue) were subcutaneously implanted into female NOG mice within two hours after the resection. The xenografted ESCC tumors (about 500 mm^3^) were harvested from the tumor bearing mice and were further implanted in female NOG mice for expansion. After three consecutive mouse-to-mouse passages, the xenograft was considered to be stabilized. To assess the therapeutic efficacy of anti-GPC1 mAb, tumor tissues were subcutaneously implanted into female SCID mice. When the mean tumor sizes reached approximately 70 mm^3^, mice were randomly divided into two groups (seven mice/group) and antibodies were administered at a dose of 10 mg/kg in 400 μl of PBS twice-weekly for 4 weeks.

### TUNEL assay

TUNEL assay (with DAPI nuclear counterstaining) for apoptosis was conducted using the ApopTag Fluorescein In Situ Apoptosis Detection Kit (Chemicon International, Temecula, CA, USA), according to the manufacturer's instructions. The images were acquired using a fluorescence microscope (BZ-9000; KEYENCE, Osaka, Japan).

### Statistical analysis

Data are shown as mean ± SD for *in vitro* experiments and mean ± SEM for *in vivo* experiments. For comparisons among three or more groups, the values were analysed by one-way ANOVA, followed by Scheffe's test. Mann-Whitney *U*-test was used for significant differences in two groups. Differences were considered significant at *P* < 0.05.

## SUPPLEMENTARY MATERIALS FIGURES AND TABLES



## Abbreviations

ADCC: antibody-dependent cellular cytotoxicity
CDC: complement-dependent cytotoxicity
ESCC: Esophageal squamous cell carcinoma
FACS: fluorescence activated cell sorting
GPC1: glypican-1
NOD: nonobese diabetic
SCID: severe combined immunodeficient
SD: standard deviation
siRNA: small interfering RNA
