# Supplementary material for: Glypican-1 targeted antibody-based therapy induces preclinical antitumor activity against esophageal squamous cell carcinoma
Source: Oncotarget. 2017 Mar 1;8(15):24741–52. doi: 10.18632/oncotarget.15799 (PMC5421884; doi:10.18632/oncotarget.15799)
Supplement: Supplementary file 1 [file oncotarget-08-24741-s001.pdf]

Glypican-1 targeted antibody-based therapy induces preclinical antitumor activity against esophageal squamous cell carcinoma

Supplementary Materials

MATERIALS AND METHODS

Immunohistochemistry

Written informed consent was obtained in all cases and the experimental protocol was approved by the ethics committees of Osaka University and National Institute of Biomedical Innovation Health and Nutrition. Surgically resected tumor tissues were obtained from patients with ESCC from Osaka University Hospital (Osaka, Japan). Non-tumor areas in the surgically resected tumor tissues from patients with ESCC were used as normal tissues. A tissue array slide of normal human tissues was purchased from Super Bio Chips (Seoul, Korea). Sections were prepared from formalin-fixed, paraffin-embedded tissue specimens, deparaffinized and rehydrated in graded alcohols. Immunohistochemical staining for hGPC1 was performed using a rabbit polyclonal anti-hGPC1 antibody (Atlas antibodies AB, Stockholm, Sweden) and a Dako ChemMate ENVISION Kit/HRP (DAB)-universal kit (K5007), according to the manufacturer's protocol (DAKO, Copenhagen, Denmark). Images were taken using a LEICA DFC495 camera attached to a LEICA DM2500 microscope (Leica Microsystems GmbH, Wetzlar, Germany).

Immunization and construction of scFv library

Total RNA were purified from HEK293 cells using an RNeasy Mini Kit (Qiagen) and cDNAs were synthesized from total RNA using a Quantitect Reverse Transcription Kit (Qiagen). To construct the hGPC1 expression vector, cDNA of hGPC1 was amplified by PCR using KOD-plus (Toyobo Co. Ltd., Osaka, Japan) with the following primers: hGPC1 forward primer 5′- CGG CCCCGCCATGGAGCTCC-3′ and hGPC1 reverse primer 5′-GGCAGTTACCGCCACCGGGG-3′. Amplified cDNA was inserted into a pcDNA3.1/V5-His-TOPO vector (Invitrogen) and designated as pcDNA3.1-hGPC1. The DNA sequence was confirmed using the ABI PRISM 3130XL Genetic Analyzer (Applied Biosystems, Foster City, USA).

To generate cell lines stably expressing hGPC1, 1×10^7 of chicken T lymphocyte-like cells were transfected with the pcDNA3.1-hGPC1 plasmid DNA by electroporation using a Bio-Rad Gene Pulser II set at 550 V and 25 μF. Transfected cells were selected using 1.0 mg/ml Geneticin (Invitrogen). FACS analyses were performed to confirm levels of hGPC1 expression in hGPC1-transfected cells.

Two-month-old Boris Brown chickens were immunized intraperitoneally (i.p.) with hGPC1-expressing chicken T lymphocyte-like cells (3×10^7 cells/0.5 ml/chicken) in an equal volume of Freund's Complete Adjuvant (Wako Pure Chemical Industries). Four additional i.p. injections of the corresponding antigen in Freund's Incomplete Adjuvant (Wako Pure Chemical Industries) were administered to chickens at 2–3-week intervals. Spleens were removed 3 days after final boosts were injected into wing veins [1].

Total RNA was extracted with TRIzol (Life Technologies) from splenocytes of chickens immunized with hGPC1-expressing chicken T lymphocyte-like cells. cDNA was synthesized with Prime Script™ II first Strand cDNA Synthesis kits (TaKaRa-Bio, Ohtsu, Japan). Antibody V-regions were amplified by PCR with KOD neo DNA polymerase (TOYOBO) using two primer pairs. CHB and CHSF were used to amplify variable heavy chains and CLSB and CLF were used to amplify variable light chains [2, 3]. The scFv linker was prepared by oligo synthesis.

Purified VH and VL fragments were assembled by PCR using a linker to make the scFv fragment. Two micrograms of scFv fragment was ligated with 2 μg of pPDS vector (accesssion number: D50401) [4] at *Eag* I and *BssH* II restriction enzyme sites. DNA was electroporated into *Escherichia coli* XL1-Blue Electroporation competent cells.

Cell panning, screening and sequence analysis

Concentrations of hGPC1-specific scFv were determined using cell panning. Cell panning was performed by BRASIL [5]. Phage-displayed antibodies were selected from a second and third round panning using FACS Calibar (BD). The base sequences of positive scFv clones were determined.

Construction of chicken-mouse IgG2a chimeric antibodies

Chicken–Mouse IgG2a chimeric expression vectors were constructed to replace the previously produced CH1/CH2/CH3 chicken–mouse IgG1 chimeric expression vector [6] with CH1/CH2/CH3 mouse IgG2a (accession number: KC295246). VH and VL of scFv were amplified

by PCR. Obtained fragments were ligated with a chicken–mouse IgG2a chimeric expression vector at *Nhe* I and *Sac* II restriction enzyme sites.

Antibody production and purification

Antibodies were prepared using the Expi293 Expression system (Life technologies). After a 96-h incubation, each culture supernatant was recovered by centrifugation and the filtration was stored at 4°C. Each culture supernatant containing anti-hGPC1 mAb was run through the Protein G Sepharose 4 Fast Flow (GE) with free fall. After washing the column with PBS, 100 mM glycine–100 mM NaCl buffer (pH 3.0) was used to elute the column. Purified antibodies were concentrated using Amicon Ultra (Millipore) to concentrations of 1–2 mg/ml.

Epitope analysis

Epitope analysis of anti-GPC1 mAb was performed according to previously reported methods with some modifications [7]. One microgram of recombinant hGPC1 (R&D Systems) was diluted with PBS and 1.0 µg of anti-GPC1 mAb or mIgG2a (Biolegend, USA) was added before incubation at 4°C for 1 h. Proteins were digested with 0.2 µg of sequencing grade modified trypsin (Promega, Madison, WI, USA) at 37°C for 1 h and/or 4 h. After digestion with trypsin, Protein G-sepharose fast flow (GE healthcare) was added before incubation at room temperature for 1 h with rotation. After washing with PBS three times, immune complexes were eluted with 0.1% formic acid. Eluted samples were diluted with PBS and the pH was adjusted to 7.0. Samples were reduced with 10 mM DTT at 65°C for 30 min, followed by carbamidomethylation with 20 mM iodoacetamide at 37°C for 30 min in the dark. Each sample was desalted using Oasis HLB cartridges (Waters, Milford, MA, USA). Desalted samples were resolved with 20 µl of 0.1% formic acid and used for analyses. LC–MS/MS analysis was performed using maXis impact (Bruker Daltonics, Inc.) coupled on-line with a Shimadzu LC 30A HPLC. A spray voltage of 4,500 V was applied. Peptide mixtures were separated on Acquity BEH C18 columns (2.1 mm × 100 mm, 1.7-µm particle size, Waters) with a flow rate of 0.2 ml/min. A linear gradient of 0% to 60% B over 50 min, 60% to 100% B over 0.1 min and 100% B over 5 min and 0% B was employed (A = 0.1% formic acid in distilled water, B = 0.1% formic acid in 80% acetonitrile). Peptides and proteins were identified by automated database searching of the Swiss-Prot protein database with the MASCOT search program (version 2.4.1; Matrix Science) and a precursor mass tolerance of 0.1 Da, a fragment ion mass tolerance of 0.05 Da, and strict trypsin specificity allowing up to three missed cleavages. Carbamidomethylation of cysteine was set as a fixed modification and oxidation of methionines was allowed as a variable modification.

Cross-reactive analysis

To construct the mGPC1 expression vector, cDNA of mGPC1 was synthesized and subcloned into a pcDNA3.4 vector (Invitrogen) and designated as pcDNA3.4-mGPC1. To determine the cross-reactivity of anti-GPC1 mAb against mGPC1, HEK293 cells were transfected with pcDNA3.1, pcDNA3.1-hGPC1 or pcDNA3.4-mGPC1. After 24 h, cells were recovered and stained with 10 µg /ml anti-GPC1 mAb or mIgG2a at 37°C for 0.5 h. Cells were washed with FACS buffer (PBS supplemented with 1% FBS and 0.1% sodium azide) for three times and stained with Fluorescein (FITC)-labelled goat anti-mouse IgG (Southern Biotech, Birmingham, AL, USA) at 37°C for 0.5 h. After washing with FACS buffer for three times, and stained cells were analysed using a FACS Canto II cytometer (Becton Dickinson, Mountain View, CA, USA) and the results were analysed using FlowJo software (Tree Star, Stanford, CA, USA).

Cell cycle assay

ESCC cells were seeded in 6-well plates at a density of 1.5×10^4 cells per well in RPMI 1640 medium supplemented with 10% FBS. NC siRNA or GPC1 siRNA were used for siRNA transfection experiments and cells were cultured in RPMI 1640 medium containing 10% FBS for 24 h or 48 h. For antibody treatment experiments, medium was removed 16 h after cells were plated and 100 µg/ml chicken–mouse chimaera anti-human GPC1 monoclonal antibody (clone 1-12) or isotype control mouse IgG2a dissolved in RPMI 1640 medium supplemented with 1% FBS and 1% penicillin–streptomycin was added for 48 h. Cell cycle analysis was performed using Cycle Test Plus DNA Reagent kits (BD Biosciences) and the FACS Canto II flow cytometer as previously described [8]. This assay was performed in triplicate.

REFERENCES

1. Nakamura N, Shuyama A, Hojyo S, Shimokawa M, Miyamoto K, Kawashima T, Aosasa M, Horiuchi H, Furusawa S, Matsuda H. Establishment of a chicken monoclonal antibody panel against mammalian prion protein. J Vet Med Sci. 2004; 66:807–14.
2. Yamanaka HI, Inoue T, Ikeda-Tanaka O. Chicken monoclonal antibody isolated by a phage display system. J Immunol. 1996; 157:1156–62.
3. Nakamura N, Aoki Y, Horiuchi H, Furusawa S, Yamanaka HI, Kitamoto T, Matsuda H. Construction of recombinant monoclonal antibodies from a chicken hybridoma line secreting specific antibody. Cytotechnology. 2000; 32:191–8.
4. Yamanaka HI, Kirii Y, Ohmoto H. An improved phage display antibody cloning system using newly designed PCR primers optimized for Pfu DNA polymerase. J Biochem. 1995; 117:1218–27.

5. Giordano RJ, Cardo-Vila M, Lahdenranta J, Pasqualini R, Arap W. Biopanning and rapid analysis of selective interactive ligands. Nat Med. 2001; 7:1249–53.
6. Tateishi Y, Nishimichi N, Horiuchi H, Furusawa S, Matsuda H. Construction of chicken-mouse chimeric antibody and immunogenicity in mice. J Vet Med Sci. 2008; 70:397–400.
7. Papac DI, Hoyes J, Tomer KB. Epitope mapping of the gastrin-releasing peptide/anti-bombesin monoclonal antibody complex by proteolysis followed by matrix-assisted laser desorption ionization mass spectrometry. Protein Sci. 1994; 3:1485–92.
8. Yokoyama T, Enomoto T, Serada S, Morimoto A, Matsuzaki S, Ueda Y, Yoshino K, Fujita M, Kyo S, Iwahori K, Fujimoto M, Kimura T, Naka T. Plasma membrane proteomics identifies bone marrow stromal antigen 2 as a potential therapeutic target in endometrial cancer. Int J Cancer. 2013; 132:472–84.

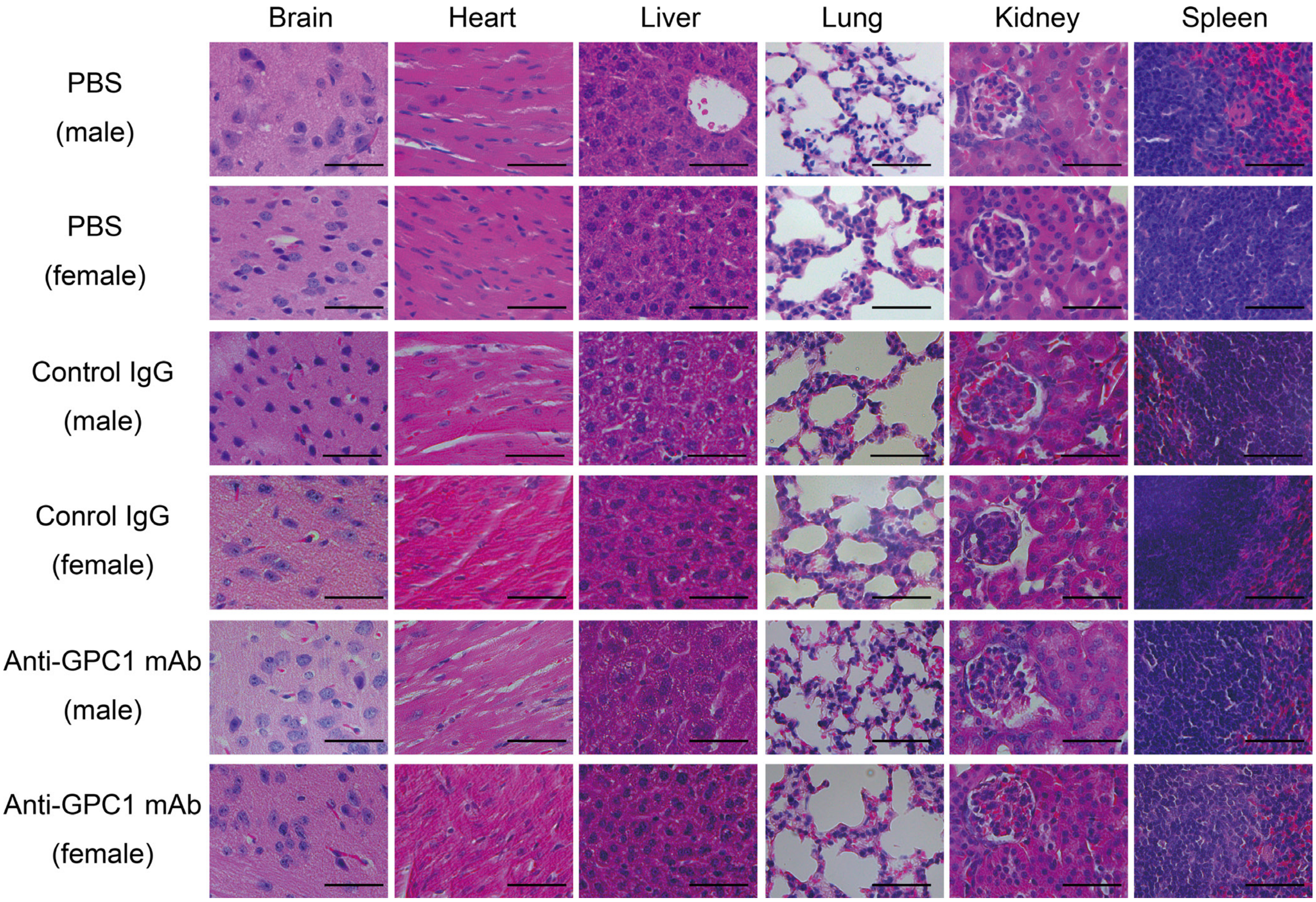

Supplementary Figure 1: Toxicology of anti-GPC1 mAb. Hematoxylin and eosin staining of paraffin-embedded mouse tissues, including the brain, heart, liver, lung, kidney and spleen seven days after administration of ctonrtol IgG or anti-GPC1 mAb. There was no evidence of anti-GPC1 mAb cytotoxicity in normal tissues.

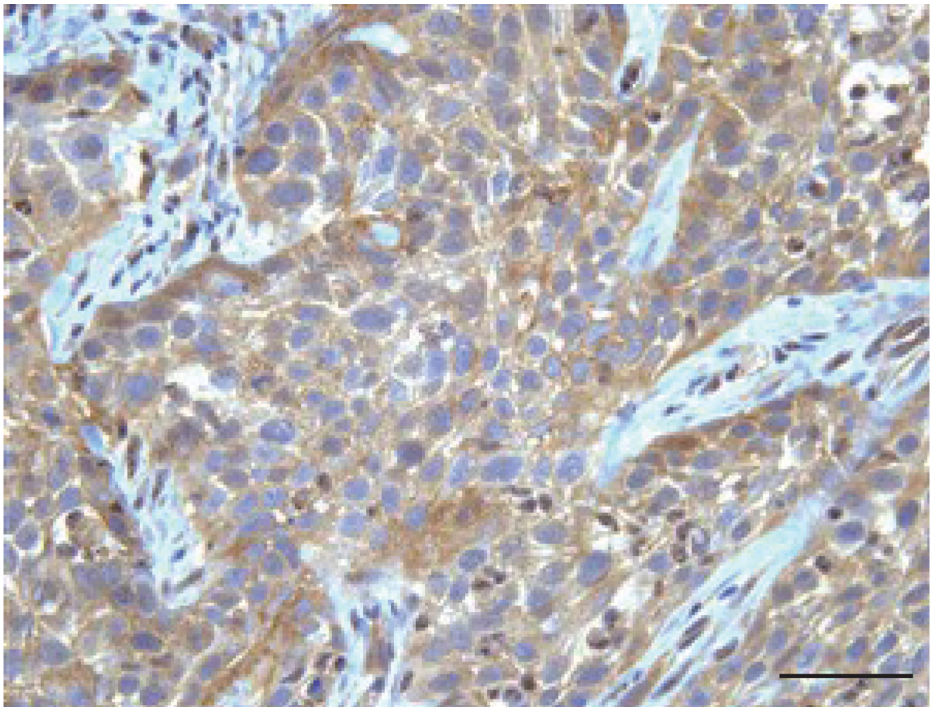

Supplementary Figure 2: Expression of GPC1 in the ESCC-8 PDX tumor tissue was analysed by Immunohistochemical staining.

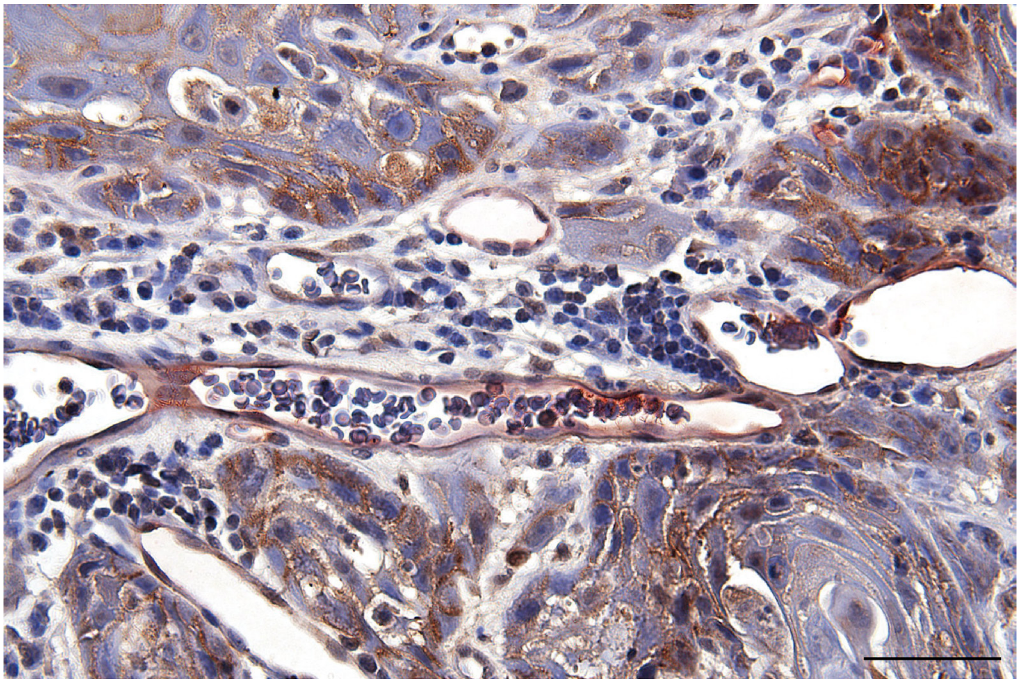

Supplementary Figure 3: GPC1 expressed on vascular endothelium cells of ESCC tissue. Immunohistochemical staining of GPC1 (DAB: brown) in ESCC tissue revealed that the expression of GPC1 was detected not only ESCC tumor cells but also CD34 positive vascular endothelium cells (Permanent Red: red) in tumor tissue. Scale bar, 50 μm.

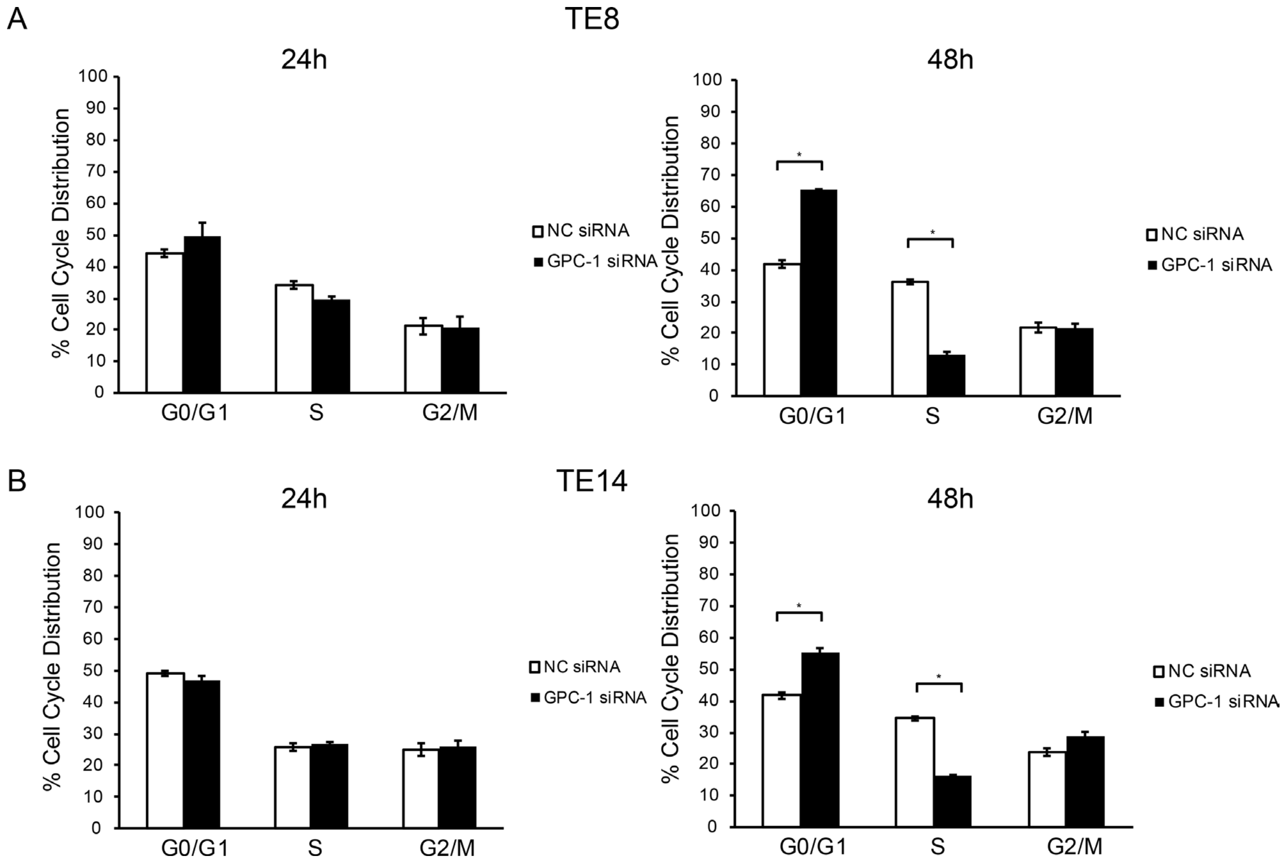

Supplementary Figure 4: GPC1 associated with cell cycle. Cell cycle analysis was performed in TE8 and TE14 cells after 24 or 48 hours of siRNA transfection. **P* < 0.001

Supplementary Table 1: Toxicological results of male mice

	Control IgG (*n* = 2)	PBS (*n* = 4)	Anti-GPC1 mAb (*n* = 3)	*p* value
WBC (10^9/l)	3.24 ± 0.03	3.05 ± 0.38	2.24 ± 0.88	N.S.
RBC (10^{12}/l)	9.48 ± 0.08	10.71 ± 0.20	9.40 ± 0.24	N.S.
Hb	14.9 ± 0.0	16.8 ± 0.4	14.3 ± 0.2	N.S.
Plt (10^9/l)	476 ± 1	335 ± 65	352 ± 105	N.S.
	**Control IgG (*n* = 6)**	**PBS (*n* = 3)**	**Anti-GPC1 mAb (*n* = 3)**	***p* value**
Alb (g/dl)	3.7 ± 0.5	3.9 ± 0.2	3.1 ± 0.0	N.S.
ALP (U/l)	139 ± 13	125 ± 11	141 ± 10	N.S.
ALT (U/l)	30 ± 3	22 ± 4	35 ± 7	N.S.
Amy (U/l)	1004 ± 71	996 ± 88	1058 ± 182	N.S.
T-Bil (mg/dl)	0.3 ± 0.0	0.4 ± 0.1	0.4 ± 0.0	N.S.
BUN (mg/dl)	22 ± 2	27 ± 4	17 ± 2	N.S.
Cr (mg/dl)	< 0.2	< 0.2	< 0.2	
Na (mmol/l)	154 ± 8	151 ± 4	149 ± 6	N.S.
K (mmol/l)	5.5 ± 0.3	4.9 ± 0.6	5.9 ± 0.6	N.S.
Glu (mg/dl)	182 ± 20	190 ± 9	186 ± 16	N.S.

Supplementary Table 2: Toxicological results of female mice

	Control IgG (*n* = 2)	PBS (*n* = 2)	Anti-GPC1m Ab (*n* = 3)	*p* value
WBC (10^9/l)	2.45 ± 0.72	2.88 ± 0.20	2.97 ± 0.57	N.S.
RBC (10^{12}/l)	9.61 ± 0.01	10.39 ± 0.17	9.98 ± 0.25	N.S.
Hb	15.1 ± 0.0	16.0 ± 0.4	15.0 ± 0.1	N.S.
Plt (10^9/l)	365 ± 118	429 ± 23	339 ± 49	N.S.
	**Control IgG (*n* = 5)**	**PBS (*n* = 3)**	**Anti-GPC1 mAb (*n* = 3)**	***p* value**
Alb (g/dl)	4.0 ± 0.2	4.2 ± 0.2	3.9 ± 0.1	N.S.
ALP (U/l)	172 ± 8	146 ± 9	155 ± 13	N.S.
ALT (U/l)	23 ± 5	24 ± 2	27 ± 3	N.S.
Amy (U/l)	864 ± 88	853 ± 100	824 ± 51	N.S.
T-Bil (mg/dl)	0.4 ± 0.1	0.4 ± 0.0	0.4 ± 0.0	N.S.
BUN (mg/dl)	19 ± 4	18 ± 2	20 ± 1	N.S.
Cr (mg/dl)	< 0.2	< 0.2	< 0.2	
Na (mmol/l)	148 ± 3	146 ± 1	147 ± 1	N.S.
K (mmol/l)	4.8 ± 0.5	4.1 ± 0.6	5.5 ± 0.4	N.S.
Glu (mg/dl)	154 ± 23	137 ± 27	179 ± 20	N.S.
